# Molecularly Imprinted Polymer (MIP) Applications in Natural Product Studies Based on Medicinal Plant and Secondary Metabolite Analysis

**DOI:** 10.29252/ibj.25.2.68

**Published:** 2021-01-10

**Authors:** Zahra Karimi Baker, Soroush Sardari

**Affiliations:** 1 Department of Horticulture, Faculty of Agriculture, Shahed University, Tehran, Iran;; 2 Drug Design and Bioinformatics Unit, Medical Biotechnology Department, Biotechnology Research Center, Pasteur Institute of Iran, Tehran 13164, Iran

**Keywords:** Molecularly imprinted polymers, Plants medicinal, Secondary metabolism

## Abstract

Characterization and extraction of plant secondary metabolites are important in agriculture, pharmaceutical, and food industry. In this regard, the applied analytical methods are mostly costly and time-consuming; therefore, choosing a suitable approach is essential for optimum results and economic suitability. One of the recently considered methods used to characterize new types of materials is MIPs. Among the various applications of MIPs is the identification and separation of various plant-derived compounds, such as secondary metabolites, chemical residues, and pesticides. The present review describes the application of MIPs as a tool in medicinal plant material analysis, focusing on plant secondary metabolism.

## INTRODUCTION

Plants have had a wide range of applications over the centuries worldwide^[^^1^^]^. People rely on plants to provide their fundamental needs such as shelter, clothing, food, and health care. These needs would enhance quickly because of urbanization, raising income, and rapidly increasing world population. In addition, plants supply raw materials for many types of pharmaceuticals, as well as for alcohol, tobacco, honey, and coffee.

In developing countries, more than half of the population benefit from the traditional animal- and plant-based medicine for their primary health care. The demand and popularity of herbal medicines are growing day-by-day. The side effects of modern drugs are the most important factor in the development of research in herbal medicines ^[^^2^^]^. Studies on traditional medicines have been a basis for the production of most early drugs, such as quinine, digitoxin, pilocarpine, aspirin, and morphine^[^^3^^,^^4^^]^.

NPs have been shown to play a significant role in drug production. They are known as one of the most valuable resources of drugs due to their biological activity and structural diversity. From 1981 to 2014 more than 50% of drugs were made from NPs, NP-derived mimetics, and semi-synthetic NPs, and between 1994 and 2014, more than a third of US FDA-approved drugs were developed or inspired by NPs^[^^5^^]^. NP-derived drugs were among the popular 35 bestselling drugs in the world in 2000, 2001, and 2002, meaning that the percentage of NP-derived drugs in these three years was 40%, 24% and 26%, respectively^[^^6^^]^.

For the best application of plant products, they must be either extracted and purified or standardized based on a lead chemical among the main bioactive ingredients contained in the plant drug. Since plant extracts contain a complex of various types of bioactive compounds or phytochemicals with different polarities, identifying and separating these compounds are major challenges^[^^7^^]^. Chromatographic and spectroscopic methods are generally applied to characterize plant metabolites. Chromatography is a commonly used technique to isolate biologically active compounds. Among such techniques are high performance liquid chromate-graphy, thin layer chromatography, size-exclusion chromatography, flash chromatography, and column chromatography. In addition, non-chromatographic methods such as immunoassay, which uses monoclonal antibodies, and phytochemical screening assays can be utilized to identify bioactive compounds. 

**Fig. 1:**
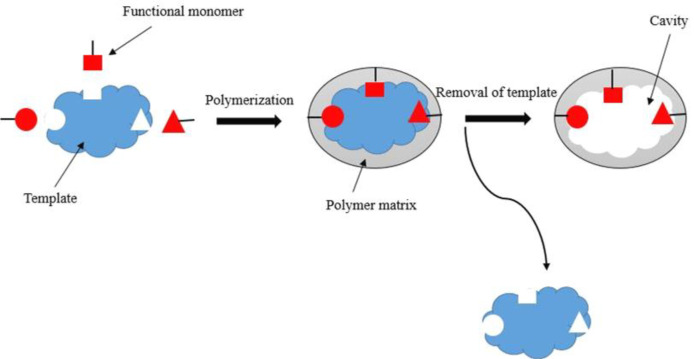
Scheme of molecular imprinting used in MIP production

MIP is a designed material that can be explained in analogy with the "lock and key model" depicted by Emil Fischer over a century ago (reviewed in^[^^8^^]^). The produced MIPs have many cavities complementary to their template molecules in shape, size, and chemical functionality, making them have a unique and predetermined selectivity towards those target molecules^[^^9^^]^. Molecular imprinting technique is used to prepare specific polymers for predetermined analyses^[^^10^^]^. In general, polymerization in the presence of a target molecule (template) with functional and crosslinking monomers causes the formation of MIPs. By removing the template from such a polymer, template-like cavity locations are formed and enable the polymer to identify the template and any template-like molecule in solution (Fig. 1). MIPs can be formed in two ways, covalent and noncovalent. In the earlier method, MIP is formed by the covalent bonds between the template and polymerizable monomers. The noncovalent approach is the formation of noncovalent bonds such as ionic interactions or hydrogen bonding between the template and the monomers. 

**Fig. 2:**
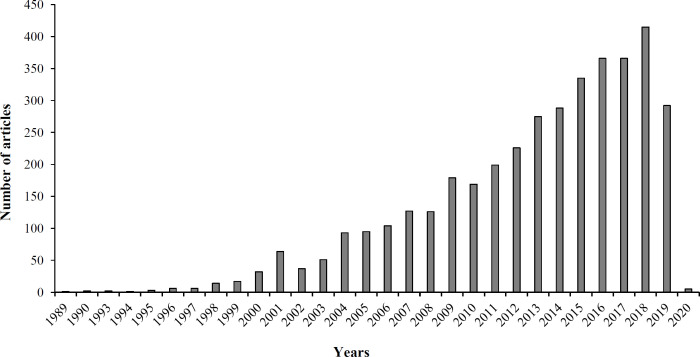
Distribution of paper found in PubMed on the subject of MIPs

**Fig. 3:**
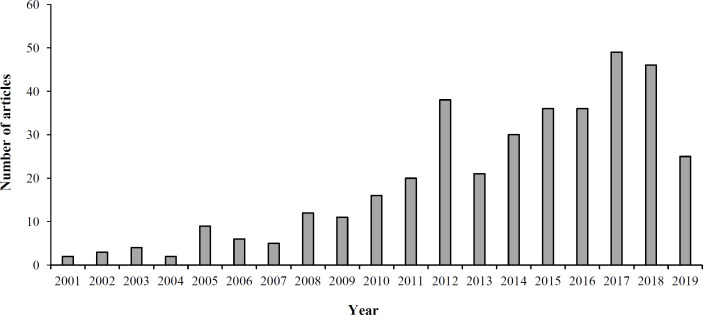
Distribution of published papers found in PubMed on the subject of plant material analysis by MIPs

**Fig. 4:**
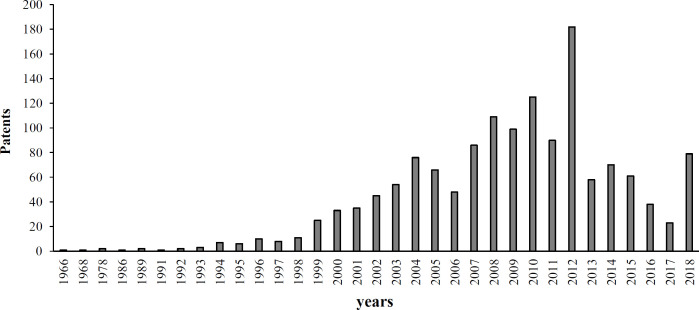
Distribution of patents in the area of MIPs, as indicated in MIP database

**Fig. 5:**
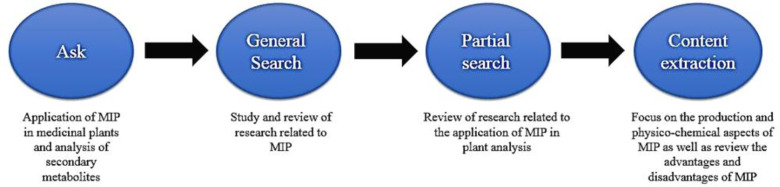
Design steps of this review

Research in the field of MIPs has received a lot of attention, as shown in the literature (Fig. 2) due to their advantages such as simple and convenient preparation, predetermined selectivity, robustness in organic solvents, acidic or basic reagents, and durability to high temperature^[^^10^^-^^14^^]^. MIPs have been exploited in cases such as the detection and isolation of specific molecules^[^^15^^,^^16^^]^, sensors^[^^17^^]^, immunoassay^[^^18^^-^^20^^]^, and catalysis as artificial enzymes^[^^21^^]^. MIPs have also been used as solid-phase extraction sorbents for the clean-up and pre-concentration of samples before determining drugs in complex biological fluids^[^^22^^-^^28^^]^, nicotine in chewing gum and tobacco^[^^29^^,^^30^^]^, and triazine herbicides in beef liver^[^^31^^]^, and water^[^^32^^,^^33^^]^. MIPs have been indicated to detect phenobarbital in the blood plasma^[^^34^^]^ and a variety of fungicides and pesticides such as carbendazim^[^^35^^]^, imidacloprid^[^^36^^]^, and organophosphate pesticides^[^^37^^,^^38^^]^ in plants. As presented in Figure 3, MIP-related research in the field of plant science has grown constantly in recent years. In the area of patents related to MIP, there is a growth pattern that has been recorded since 1966 and is shown in Figure 4. These data sources were applied in the preparation of the current manuscript, and Figure 5 provides a schematic illustration of the design steps in this study.

**MIP application in plant analysis**

The technique involving MIP usage is a fast, low-cost one and has wide applications in identifying compounds in the field of biology. In this regard, MIPs made with plant metabolites are also a useful method for identifying and extracting plant compounds. Some examples of MIP application in the field of plant material analysis have been described below.

Identification and quantitation of gallic acid, a phenolic secondary metabolite, in medicinal plant extracts and food ingredients such as orange juice has been a recognized issue. The expanded pipette-tip molecularly imprinted silica monolithic HPLC technique has been used practically for tracing gallic acid in orange juice samples and shown the reliability of this method^[^^39^^]^. The accuracy of this method was examined by the extraction performance of spiking samples at three concentrations (0.05, 0.50, and 2.00 mg/L) with a standard solution of gallic acid. The values of gallic acid in the spiked orange juice samples were in the range of 95.6–100.5% and 92.0–97.0%, respectively. The results displayed that the usual method was impressive and reliable for monitoring gallic acid in orange juice samples. No interposition with other constituents of orange juice was observed on the surface of MIP monolith^[^^39^^]^.

In the analysis of solutions containing plant materials, MISPE has demonstrated that the components in the TCM extracts are nearly completely adsorbed onto the MIPs^[^^40^^]^. The extraction performance using this method for a terpenoid substance called Kirenol was 80.9%, and the recovery of the spiked solution was 91.5% ± 3.2 (n = 3). According to these findings, the MISPE method is suitable for the enrichment and determination of Kirenol in TCM and for the selective clean-up of plant extracts. In a study conducted by Ghasemi *et al*.^[^^41^^]^, imprinted membrane was employed to enrich paclitaxel from yew tree extract up to 48%. Due to simplicity and cost-effectiveness, this method can be an alternative to difficult and expensive approaches in identifying paclitaxel^[^^41^^]^. MIPs have also been successfully used to identify polyphenols in plant extracts^[^^42^^]^ and to extract glucosamine from chicory roots with promising results ^[^^43^^]^.

MMIP for coumarin (MMIP-coumarin) has been applied to identify and extract coumarin from food and plant samples^[^^44^^]^. Quercetin and kaempferol are among the flavonoids in Ginkgo leaves, and the MIPs designed for these substances can trap them in Gingko leaves extract^[^^45^^]^. MIPs designed for the extraction of selective PA from *Homalomena occulta* and *Cynomorium songaricum* extracts were used with well performance^[^^46^^]^.

MIP has also been successfully used to extract chicoric acid from *Cichorium intybus*. This MIP has shown high selective ability when it is utilized against the template’s structural analogues, including CA, caftaric acid, and chlorogenic acid^[^^47^^]^. Besides, MIP has been utilized to separate epigallocatechin gallate in tea extract. A high purity of epigallo-catechin gallate has been obtained by using this method, and the mean recoveries of this compound was 87.42%. As a result, MIP method could be suitable for extracting and purifying the aforementioned plant compound^[^^48^^]^. Emodin is extracted from the alcoholic extract of *Rheum palmatum L.* using MIP with a purity of 95%.^[^^49^^]^. MIP can serve as an adsorbent for the extraction of quercitrin myricetin, and amentoflavone in *Chamaecyparis obtuse* extract^[^^50^^]^. Using 3 g of adsorbent under optimized conditions, 0.45 mg/g of quercitrin, 0.18 mg/g of myricetin, and 0.12 mg/g of amentoflavone were obtained. According to the results of that study, the method used to separate the the metabolites has a small deviation error and is also very selective and repeatable. In one study, PA was applied as a model for the construction of MIP, which utilized to identify compounds in *Melissa officinalis*. The MIP was unable to determine the gentisic acid, and the recovery power of the syringic acid was low (16.5%). However, other phenolic acids such as protocatechuic acid, gallic acid, p‐hydroxybenzoic acid, and vanillic acid were extracted with more than 56% yield^[^^51^^]^. A study had been performed to determine the possibility of extracting caffeine and theophylline from green tea using MIP^[^^52^^]^. Two types of MIP were designed with two different patterns, one was caffeine-theophylline mixture, and the other was pentoxifylline-theophylline mixture. The results revealed that MIP could be exploited to extract caffeine and theophylline from green tea solutions.

MIP has been used to separate the sinomenine in *Sinomenium acutum*. As a result, almost pure sinomenine was obtained by MIP. In addition, by applying the MIP designed for sinomenine, it is possible to identify the sinomenine in biological materials. Besides, results revealed that MIP technique can be widely used in the fields of plant metabolite extraction and biopharmaceutical analysis^[^^53^^]^. Table 1 shows examples of plant compounds identified by MIP. In our previous work, MIP was used to detect hyoscyamine in *Lactuca scariola*^[^^54^^]^. According to the results, the unique three-dimensional, highly-ordered photonic hydrogels would be obviously bound in response to the specific atropine molecular recognition process, and the response would be directly transferred into visually perceptible optical signal that could be detected by the naked eyes^[^^54^^]^. 

**Production and physico-chemical aspects of MIP**

***Design and production of MIPs***

MIP production is easy and inexpensive and does not require sophisticated tools. There are ways to produce MIPs having one basis. Most of them need a crosslinker, solvent, and surfactant. One way to produce MIP is to use sol-gel concept; some researchers have employed this technique to make MIP^[^^39^^,^^49^^]^. The surface imprinting technique has been used to produce MIP-based sorbents with a polymer layer coated on the Fe_3_O_4_ surface^[^^44^^]^. In 2018, for the first time, scientists reported the successful production of a paclitaxel made from polysulfone membrane, which improved the separation of such materials^[^^41^^]^. In preparing MIP to absorb polyphenols, the material obtained from 4VP as a functional monomer, ethylene glycol dimethacrylate as a crosslinker, water/methanol as a solvent, n-heptane as an oil, and Span 80 as a surfactant showed better performance to other forms of MIP in absorption process^[^^42^^]^.

***Washing step***

Washing of pattern monomers (target molecule) from MIP is one of the most essential steps in the MIP production process because presence of pattern molecules in the MIP interferes with binding of the desired composition and causes errors in the work. It is important to choose the best washing solvent, i.e. the one that will wash the monomer from the MIP and does not damage the MIP. Researchers have tested various solvents to select best functional ones. High purity hexane has widely been used as a solvent for washing MIP. The best recovery came when methanol:acetic acid 90:10 was applied for washing. Adding acetic acid makes it easier to remove the template^[^^39^^]^. In a study, acetonitrile (used to load the samples) and water were utilized as washing solvents. Less than 5% of the template was washed with water or acetonitrile alone^[^^51^^]^. 

***Physical properties***

*Binding capacity*

The binding capacity depends on factors such as the quality of the prepared MIP and the amount of porosity to bind the target material. There is a competition between the desired compound and similar compounds to bind the MIP. In a study conducted by Karasová *et al*.^[^^51^^]^, the connection of target compounds to the designed MIP was very appropriate so that the MIP had the highest connection capacity with PA (34.7 µg/g of MIP) and gallic acid (29.5 µg/g of MIP). It has been shown that MIP not only can separate phenolic acids from other substances in the extract but also can separate phenolic acids from each other^[^^55^^]^. Moreover, the connection capacity of MIPs is more than NIPs^[^^44^^]^. In 2016, the researchers generated two types of MIP, one using methacrylic acid and the other with acrylic acid^[^^56^^]^. In the MIPs with methacrylic acid, connection capacity was higher than the MIPs using acrylic acid. Methyl groups in methacrylic acid may repel other groups, making methacrylic acid MIP more porous. As a result, methacrylic acid MIP has a greater ability to absorb quercetin than acrylic acid MIP. The best binding capacity and specific surface area of this research was 392.08 µg/g and 167.899 m^2^/g, respectively, which is pertained to methacrylic acid MIP. There has been MIPs designed to bind to chicoric acid with a high binding ability to this substance^[^^47^^]^. MIP has a high absorption capacity to its template, no matter how complex is its template matrix and has the ability to separate that material^[^^49^^]^. After synthesizing MIP, Dong *et al*.^[^^57^^]^ conducted an experiment to evaluate the combined affinity of MIP and non-imprinted blank polymer to (−)-ephedrine. The results showed that MIP had a higher binding capacity for (−)-ephedrine than non-imprinted blank polymer. 

**Table 1:** Samples with plant origin analyzed by MIPs

**Template**	**Sample**	**Metabolites**	**Reference**
Gallic acid	orange juice	Gallic acid	^[^^39^^]^
Protocatechuic acid	*Melissa officinalis*	P-hydroxybenzoic acid	^[^^51^^]^
Nicotine	*Nicotiana rustica* (tobacco)	Nicotine	^[^^30^^]^
Pharmacophoric flavonoids	*Ginkgo biloba*	Flavonoids	^[^^45^^]^
Phenolic acids	*Salicornia herbacea*	Phenolic acids	^[^^55^^]^
Kirenol	*Siegesbeckia pubescens*	Kirenol	^[^^40^^]^
Osthole	*Libanotis Buchtomensis*	Osthole	^[^^58^^]^
2,4-dinitrophenol	*Taxus baccata*	Paclitaxel	^[^^41^^]^
Coumarin and 7-hydroxycoumarin	*Cinnamomum verum, Matricaria chamomilla, Lavandula, dried archangel*	Coumarins	^[^^44^^]^
Glucose and fructose	*Malus domestica*	Glucose and fructose	^[^^60^^]^
Polydatin	Red wine	Polyphenols	^[^^42^^]^
Glucosamine	*Cichorium intybus*	Glucosamine	^[^^43^^]^
Protocatechuic acid	*Homalomena occulta* and *Cynomorium songaricum*	Derivates of p-hydroxybenzoic acid	^[^^46^^]^
Chicoric acid	*Chicorium intybus* L.	Chicoric acid	^[^^47^^]^
Epigallocatechin Gallate	*Camellia sinensis*	Epigallocatechin gallate	^[^^48^^]^
Emodin	*Rheum palmatum *L.	Emodin	^[^^49^^]^
Betulin	*Platanus *sp	Betulin and Betulinic Acid	^[^^59^^]^
Flavonols and Flavones	*Chamaecyparis obtusa*	Flavonols and Flavones	^[^^50^^]^
Quercetin	*Ginkgo biloba*	Pharmacophoric flavonoids	^[^^45^^]^
Quercetin	*Caragana Jubata*	Anti-EGFR inhibitors	^[^^61^^]^
Quercetin	*Ginkgo biloba*	Quercetin and kaempferol	^[^^45^^]^
Sinomenine	*Sinomenium acutum*	Sinomenine	^[^^53^^]^
Podophyllotoxin	*Sinopodophyllum emodi*	Podophyllotoxin	^[^^62^^]^
Caffeine-theophylline and pentoxifylline-theophylline	*Camellia sinensis*	Caffeine and theophylline	^[^^52^^]^
Matrine	*Sophora flavescens*	Matrine	^[^^63^^]^
Protocatechuic acid	*Melissa officinalis*	Derivates of p-hydroxybenzoic acid	^[^^51^^]^
Esculetin	*Fraxinus excelsior*	Esculetin	^[^^64^^]^
(−)-ephedrine	*Ephedra sinica*	(−)-Ephedrine	^[^^57^^] ^
Harmine	*Peganum nigellastrum*	Harmin	^[^^65^^]^

**MIP as an analytical technique**

***Difference between MIP and NIP***

The NIP is a structure, which in its production process, a pattern has not been used. The contrast between making MIP and NIP is to add a pattern that makes them slightly different. This discrepancy makes the MIP more capable of absorbing the desired compound than the NIP. Images captured with Atomic Force Microscopy and scanning electron microscope taken from MIPs and NIPs show differences in the size distribution of MIP and NIP particles^[^^56^^]^. Some dissimilarities were perceived between the NIP and MIP in terms of selectivity. The basic interplay involved in the retention is ionic interaction, hydrogen bonds, and hydrophobic patches in addition to shape formations in cavities. Therefore, these interactions could keep various molecules on the two polymers types, but MIP is more selective and has more capacity than NIP^[^^43^^]^.

***Economic aspects of MIP recycling***

One of the important abilities of MIPs is their reusability. This is a very important advantage in saving time and money. There has also been research into the MIP recycling. For instance, the ionic liquid-based molecularly imprinted anion-exchange polymer is used to separate PA, CA, and FA in *Salicornia herbacea* L. extract is effectively recyclable. The recovery efficiency of PA, CA, and FA by IMAP over four rounds of usage was 90.1–84.7%, 95.5–87.2%, and 96.6–88.3%, respectively^[^^55^^]^. 

A study of MIPs for the selective extraction of coumarins from plant samples showed that after 10 recovery cycles, MIP could still be used to isolate substances; hence, a decrease in MMIP-7-hydroxycoumarin uptake capacity of less than 5% was reported^[^^44^^]^. Molecularly imprinted membrane made by Ghasemi, *et al*.^[^^41^^]^ was applied at three reconstruction cycles in a selective identification mode. Acceptable recycling has been observed in an MIP designed by He *et al.*^[^^58^^]^. The regeneration cycle is repeated six times, with the absorption capacity reported to be 94.9%, indicating the strength and stability of the MIPs. Decreased absorption capacity can be due to the blockage of some MIP cavities after recovery or their disappearance during washing. The recycling power of NIP is also examined, which according to the results obtained, the composition of NIP remains almost unchanged because the recognition of NIP is non-specific, and the effect of washing or regeneration is negligible^[^^58^^]^.

**MIP advantages and disadvantages**

MIPs have many benefits as an analytical tool in detection, separation, and isolation. These advantages make the use of MIPs promising for the identification and extraction of plant compounds, and given these benefits, a bright future is foreseen for them. One of the advantages of this method is its low cost and very low consumption of experimental materials^[^^39^^]^. This method is fast and cost-effective. The ability to reuse and recycle MIP is one of the highlights^[^^30^^]^. The superior power about MIPs is that they can detect and absorb specific molecules^[^^39^^,^^48^^,^^56^^,^^58^^,^^60^^]^. MIPs have shown high binding and adsorption capacity of the target molecule^[^^46^^,^^58^^]^; Moreover, MIPs have a high durability against various conditions^[^^49^^]^. Using MIPs can be a convenient and practical way to identify and isolate plant metabolites. According to research, they were used to identify different substances in plants. This technique can be very useful in herbal research and pharmacy due to its many merits such as simplicity, affordability, and high sensitivity. MIPs have downsides that limit their use, and such barriers have prevented their remarkable progress. Disadvantages can be attributed to the length of analysis time. MIPs are also made with a large amount of template, a number of imprint molecules may remain in the polymer. Hence, there could be a disruption in the analysis^[^^52^^]^. 

## Conclusion

In general, MIPs are highly applicable to plant and natural product analysis. As a final remark, it should be noted that although MIPs are difficult to optimize, their versatility and sensitive nature make them suitable for analysis in terms of identification and purification.

## CONFLICT OF INTEREST.

None declared.
